# The History of Inpatient Care in German Departments Focussing on Natural Healing

**DOI:** 10.1155/2013/521879

**Published:** 2013-05-08

**Authors:** André-Michael Beer, Bernhard Uehleke, Karl Rüdiger Wiebelitz

**Affiliations:** ^1^Department of Naturopathy, Blankenstein Hospital, Im Vogelsang 5-11, 45527 Hattingen, Germany; ^2^HfG Hochschule für Gesundheit und Sport, Germany Hochschule für Gesundheit und Sport, Vulkanstr. 1, 10367 Berlin, Germany; ^3^Clinic for Children and Adolescents, Prignitz Hospital, Dobberzinerstrasse 112, 19348 Perleberg, Germany

## Abstract

We describe historic developments of inhouse facilities for natural healing in this paper, which were mainly located in German speaking regions. The naturopathic movement is a relabeling of the hydropathic movement in Germany, which was supported by a considerable proportion of the population in Germany during the mid 19th century. Due to the fact that hydropathic treatments were provided by nonmedical healers, discriminated as “quacks”, there was continuous hostility between hydropathy/naturopathy and medicine. However, among the many establishments providing inhouse treatment for acute and chronic diseases over weeks there were some which were controlled by medical doctors in the 20th century and some which were implemented by government. In many of the establishments there were approaches for measuring usefulness of the treatments, some of which have been initiated explicitly for that purpose.

## 1. Introduction

Natural healing uses the philosophy of naturopathy with a focus on a nature-orientated healthy life style. Naturally healing methods are also applied in the therapy of disorders and in rehabilitation. They are used in self-care often recommended by nonmedicals and medical practitioners in ambulant settings. There are also special clinics, hospitals for inhouse treatments. We present the historical development of naturopathy with focus on inpatient natural healing with regard to present and future statuses. The asclepion of the ancient Greek temple facilities might be referred to as an early precursor of inpatient treatment with a programme similar in many aspects to that of contemporary natural healing. Apart from the medicinal water applications a dormitorium was also in use for inducing a healing sleep—some similarity might be seen with modern meditation [1].

A dogmatically independent and new development contrary to the medicine of the time [2] was constituted as late as the beginning of the 19th century; but the name for this programme was initially hydropathy or in German “Wasserheilkunde.” However, in the 18th century there was already an increased interest in medicine with a view towards mild hippocratic approaches of healing, inclusive internal and external water applications, healthy food and physical exercises, and avoidance of dangerous and rigorous interventions; all this was related sporadically to the term “medicina naturae” [3].

The hydropathic movement developed and was driven mainly by medical laymen [4]. About the mid 18th century, this romantic hydropathical movement changed its name to a more positive term, that is, “Naturheilkunde” (naturopathy, art of natural healing). Exercise, nutrition, and later other natural healing methods like herbal therapy were added to cold water. Natural treatments were provided by medical and nonmedical healers as well as in self-help groups in both in- and outpatient settings. This was in contrast to the established medical services—the conventional balneology is included, with its focus on special and locally available spa treatments. During the second half of the 19th century spas or hospitals specializing in natural healing developed worldwide.

The books and quarterly water journal of Eucharius Ferdinand Christian Oertel (1765–1851) and also his first association with hydropathic health were important for the dogmatic and medicine hostile development. Similar to other systems and disciplines of complementary medicine a very broad support of the population was the reason for its growth despite stakeholders in medicine and governments.

The aim of the paper is to describe the subsequent development of the relationship between naturopathy and conventional medicine with special regard to inpatient treatment of seriously ill patients.

## 2. Methods

The content of this paper relies on the literature review in AR96 (Deutsches Ärzteblatt), AZ72 (GLOBAL Health), BA70 (BIOSIS Previews), CB85 (AMED), CC00 (CCMED), CCTR93 (Cochrane Central Register of Controlled Trials), CDAR94 (Database of Abstracts of Reviews of Effects), CDSR93 (Cochrane Database of Systematic Reviews), CV72 (CAB Abstracts), DAHTA (DAHTA-Datenbank), DD90 (Derwent Drug File), EA08 (EMBASE Alert), ED93 (ETHMED), EM47 (EMBASE), GA03 (gms), GM03 (gms Meetings), IA70 (IPA), II78 (ISTPB + ISTP/ISSHP), INAHTA (Health Technology Assessment Database), IS74 (SciSearch), ME60 (MEDLINE), MK77 (MEDIKAT), NHSEED (NHS Economic Evaluation Database), SM78 (SOMED), and ZT00 AnimAlt-ZEBET) using “NATUROPATH%## AND INPATIENT#” and “STATION? AND NATURHEIL?” for the search (with case insensitivity). The searches produced 48, respectively, 89, together 137 hits. After elimination of double or multiple hits of the same source 85 hits remained. 39 papers were excluded from further evaluation because they did not examine the naturopathic inpatient treatment according to their titles and abstracts. Another 25 sources were excluded by full-text analysis for the same reason or because they were only concerned with the naturopathic inpatient treatment of a special condition or disease. The remaining 11 papers, supplemented by own additional literature, which included already 9 of the 11 papers found by the systematic search, were used to elaborate the content of this paper. A previous published historical review of the development of naturopathic inpatient treatment was not found.

## 3. First Naturopathic Inpatient Facilities

The first and famous cold-water establishment was developed by the farmer Vinzenz Prießnitz (1799–1851) in Gräfenberg/Freiwaldau in Silesia [5]. Prießnitz opened his cold-water spa in 1822. He treated 45 patients in 1829, 500 patients in 1837, and two years later there were already 1700 patients, among them 120 physicians. The diagnoses of his patients during the years 1829 to 1839 are analysed and descriptively reported by Sajner and Křížek [6].

Some time later, hydrotherapy was extended by certain forms of nutrition therapy. Johann Schroth (1798–1856), a schoolfellow of Prießnitz, was striving for the concept of the Prießnitz's water establishments and founded his own cure establishment in the nearby Niederlindewiese, where, along with warm water applications, he introduced a fasting cure with alternative days of drinking large amounts of wine or water fluid and days without drinking at all [7]. Later on, ideas of vegetarianism were incorporated in the movement of naturopathy.

The term “Naturheilkunde” (naturopathy) was initially introduced by the forest geometer J. H. Rausse alias Heinrich F. Francke (1805–1848). Later the concept was extended in 1849 by the Bavarian physician Lorenz Gleich (1798–1865), a radical representative of hydropathy and naturopathy and in strong opposition to conventional medicine [8, 9]. He could also accommodate some patients in and alongside his hydropathical facility in the south of Munich. Apart from hydrotherapy, there were also nutrition and physical exercise therapies applied.

The further development included a systematic use of light, air, and the sun introduced by the so-called “sun-apostle” [10], Arnold Rikli (1823–1906) [11] and later on by the open-air fanatic, Adolf Just (1859–1936) [12]. They applied the whole spectrum of the natural cure factors in their air-cottage parks: Adolf Just is today known as a rediscoverer of internal and external treatments with “Heilerde” (healing clay) [4].

Hydropathical-orientated naturopathy could not develop to a standardized and widely accepted level until the 1870s, with an exception of a broad active movement of naturopathy in Saxony, where the physician Heinrich Lahmann (1860–1905) resumed the leadership of Zimmermann's notable establishment of true naturopathy in Chemnitz in 1886. However, two years later Bergmann opened his own facility for naturopathy (Weißer Hirsch) near Dresden.

## 4. The Kneipp Movement

A true renaissance of naturopathy all-over Germany and beyond started with the Catholic priest Sebastian Kneipp (1821–1897). His bestseller “My Water Cure” (1886) was intended to reduce personal provision of the treatment. However, the opposite happened (in 1889 over 2,600, 1892 over 12,000 patients in Wörishofen). Using donations by his patients, Kneipp established several hospitals “Sebastianeum,” “Kneipp's healing facility for children”, and the “Kneippianum.” The last was managed like a hospital by the Kneipp physician Alfred Baumgarten (1862–1924) who started in 1894.

The first Kneipp association was established in Wörishofen in 1891 and later named “Stamm-Kneipp-Verein” (Original Kneipp Society). The development of other local societies followed rapidly. The “Verein der Ärzte Kneippscher Richtung” (the Society of Kneipp Physicians) (later “Kneipp Ärztebund”, Union of Kneipp Physicians) was established in 1894.

Kneipp is regarded as the “reformer of hydropathy” due to his recommendation of a much shorter cold water stimulus, which led to a better initial reaction and better long-term results. Kneipp introduced affusions (from a watering can without sprinkling head or from a wide mouthed rubber tube). Kneipp did not only—as could be assumed from the title of his book “My Water Cure”—focus on hydrotherapy (Part 1 of his book), but also combined his water therapy with herbal medicine. This was heavily criticised by other hydro-therapists and naturopathists, while they assumed a “bad compromise” that would detract patients from their strict self-healing process and soften the cure too much. Additionally, Kneipp combined water and herbs with exercise, diet, and guidelines to healthy and happy life, specially in his further books “Thus Shalt Thou Live” (1889) and “My Will for Healthy and Sick” (1894).

Diseases were described very simply and clearly: they originate either because of dysfunction of blood composites or circulatory disturbance. Accordingly, a therapy succeeded due to liquidation of obstruction or here of aroused harmful substances and their discharge and secretion. The cause of sensibility and susceptibility for getting sick is the absent self-purification.

Regarding the diet, Kneipp appeared not to be radical and puritan but rather praised the inartificial plain fare; clothing should not be restrictive; omitting footwear prevents driving blood up in a harmful way. The skilled weaver was against woollen clothes touching skin directly and preferred linen because of its rub effect. A fresh and unspoiled air was important as well.

The “five columns,” which nowadays are accredited to Kneipp, do not originate directly from him. They are formulated after 1950 by the Kneipp physician Josef H. Kaiser, and they include water, nutrition, physical exercise, herbal treatments, and “Ordnung” (balance of life or today: mind and body).

Kneipp was at the time reluctantly noticed by the conventional academically minded medical establishment and, for example, ignored by Wilhelm Winternitz (1834–1917), who considered himself a successor of the long, since deceased, Prießnitz. He was also looked down upon by Ferdinand v. Ziemssen of Munich hospital. In fact the academic writings of Professor Winternitz from 1877 never garnered such public attention as the books of the “simple” priest.

In 1889, the Jordan bath—the first Kneipp bath outside Wörishofen—was opened under leadership of Dr. Johann Nepomuk Stützle (1858–1938). Though numerous Kneipp establishments were founded, only some of them are still in operation like the one at Brixen (now Italy), founded in 1890 by Otto V. Guggenberg (1848–1914).

Another core area of naturopathy was Dresden and Saxony. The physician Paul Kadner (1818–1868) had opened the first diet cure establishment which had 20 beds as early as 1861. Later his brother-in-law Felix Klees (1832–1899) continued with focus on the Schroth cure. The physician Heinrich Lahmann (1860–1905), who served as head of a big naturopathic facility in Chemnitz before, established the leading sanatorium in the spa town Weißer Hirsch at Dresden. Over 2000 patients were treated there in 1900. Several other health hospitals were situated in the neighbourhood and in the area around Dresden, part of which operating under control of physicians [13].

At the turn of century, greater political pressure was exerted upon medicine to use naturopathy to a greater extent. This appeared under the newly established branch of medical science “physical-dietary medicine” which ranged from hydrotherapy, massage, and remedial gymnastics to diet. Neither the medicinal herbs introduced by Kneipp nor some further specific ideas and treatments of naturopathy were recognized by the medical approach.

## 5. First Academic Naturopathy

Ernst Schweninger (1850–1924) [14, 15] was appointed professor for dermatology after his successful treatment of Bismarck in 1884–1900, and during 1900–1906, he was head of the first German hospital of naturopathy in Berlin-Groß-Lichterfelde associated with the Charité [16].

Schweninger treated 8, 359 patients in Groß-Lichterfelde, administering 262, 118 treatment days. Among those there were 479 consumptive patients (i.e. mainly tuberculosis), 264 acute joint-rheumatic patients, 219 gastro patients, 210 diphtheria cases, 165 scarlatina cases, 155 heart cases, 141 syphilitics, 129 gonorrhoea cases, 113 eczema cases, 104 pneumonia cases, 72 red murrain cases, 45 rubeola cases, 34 psoriasis cases, 27 typhus cases, and 16 pertussis cases [17].

The physician Georg Hauffe (1872–1936), a former assistant of Schweninger, devoted himself subsequently to Groß-Lichterfelde—a municipal hospital for physical-dietary therapy and in particular hydrotherapy.

Additionally, since 1901, there existed a hydrotherapic facility at the Charité under Professor Ludwig Brieger (1849–1919), who later also held the chair for general therapy [18]. Franz Schönenberger (1865–1933) [19] was proposed by the Prießnitz society as successor to Brieger, and though opposed by the faculty he was nominated professor and head of the hydrotherapic facility of the university. At the time, this consisted of a polyclinic with surgeries and a small hospital department with just 20 beds. 25,000 patients were medicated within nine years. Most were from a poverty stricken background from the north of Berlin. About 100 women and 50 men were treated in the bath facilities daily. 250,000 water applications were applied according to the prescriptions of physicians during that time. 56,000 patients were visiting the so-called “electrical department.” The department for Swedish remedial gymnastics and massage was reestablished in 1921, and 4,500 applications were provided within eight years.

Paul Vogler (1899–1969), a former assistant of Schönenberger, followed Schönenberger 1941–1965 [20]. The professorship for physical-dietary therapy and physiotherapy in Berlin (East) was then assigned to Herbert Krauß (1909–1991) in 1965. E. Conradi was the successor of Herbert Krauß from 1969 to 2001.

In 1924, a second university department for naturopathy was opened at the Friedrich-Schiller University in Jena. Ernst Klein [21] was the first professor for naturopathy.

The first teaching hospital of naturopathy with 75 beds, the Prießnitz hospital in Berlin Mahlow, was established in 1927 and affiliated with the natural healing department of Professor Schönenberger. The public acceptance was so great that the patients waited several months for admission. The therapeutic measures described by Brauchle [18] included uncooked vegetarian food according to Bircher-Benner (1867–1939) [22], cold water hydrotherapy according to Prießnitz and Kneipp, warm applications according to Schweninger, and air and sun baths according to Rikli [10, 11] and Lahmann [23], as well as the Schroth cure in a moderate variant. Additionally, the fasting cure, massage, and gymnastics played a significant role. The successor was Alfred Brauchle (1898–1964) who stayed there until he left for Dresden.

## 6. “New German Medicine”

Naturopathy was abused under the state dictatorship of national socialism during the third Reich and was part of the intended “New German Medicine.” Different medical areas such as naturopathy, homeopathy, and biochemistry by Schüßler, the last mostly due to the impressive number of members in the nonmedical societies, were put together under “biological methods.” The anthroposophy was, however, labelled as “degenerated” and forbidden.

Ernst Klein in Jena was dismissed in 1933. The successor of Klein was the party politically active Karl Kötschau (1892–1945). The department of true naturopathy at the University of Jena was transformed under his leadership from a polyclinic for naturally healing systems into the “Clinic and Polyclinic for Biological Medicine” [24].

Among a number of initiatives at the Reich's level that had to facilitate the prevalence of naturopathy the Reich's working group of nature physicians (1935) voted for the establishment of a hospital for naturopathy as part of the medical university hospital in Erlangen.

This project was implemented not in Erlangen but at the Rudolf Heß hospital (former Johannstädt hospital) in Dresden. Restructuring the former huge hospital with about 1000 beds was completed in 1935, and the experiment of Dresden could start. Louis Redcliff Grote (1886–1960) was nominated as the head of the hospital for internal medicine with about 300 beds. In parallel, the hospital for naturopathy was subordinated to Brauchle and comprised after its extension about 250 beds [25, 26].

Hydrotherapy, massage and gymnastics, and air and sun baths, as well as the upcoming psychotherapy (according to Coué und Wetterstrand) were applied in Brauchle's Department. Clinical visits were arranged during daily air baths. Regular meetings of medical staff were held. They were therapeutically supervised by a physician of the ward of the true naturopathic department and assisted by an internist assigned for diagnostic advisory [18, 27]. Both departments had daily rotating admission shifts. The people treated ranged from patients with internal diseases as well as patients of conservative gynecology, orthopedics, neurology, and dermatology. Brauchle and Grote performed associated visits at the common ward which was the core place for critical dialogs regarding specific cases.

The treatment was purposely aligned with simplicity and strict manageability in order to evaluate the effects of naturally healing systems under the preconditions of a large-scale hospital. After the initial examination, a treatment program was developed by the team of true naturopathic physicians. Medication was given only with the objective to improve the prospective effect of physical-dietary therapy.

Relaxation techniques were administrated to all patients. Regular lectures of the physicians to public health, detailed final meetings with proposals for individual arrangements, and daily hydrotherapy were also available for the inpatients.

According to Krauß [27] the duration of stay in the hospital for true naturopathy lasted 22 days, in the department of Grote 21 days. The average costs of medication were 35 Pfennig per day at Grote and between 4–6 Pfennig per day at Brauchle (skin oils and herbal teas included).

The difference of total costs was due to different usage of medication, number of nurses, and therapeutic staff. It was also dependent on complexity of laboratory-technical work. No documentation of daily personnel costs during the “experiment of Dresden”, which reassesses the methods of naturopathy [28], is available.

In accordance with the political development Brauchle elaborated at the beginning of the forties his psychological collective treatment into a form of mass suggestion (“Massensuggestion”) [29].

Death was declared by Brauchle as “best cure” for patients when naturopathic methods could not help them [28]—a cynical point of view.

After 1942, the deteriorating general situation during the second world war stopped the harmonic cooperation of Brauchle and Grote [30].

The occupation of naturopathy by national socialism recharged the beginning of academic recognition which naturopathy gained at 1920 (chair for naturopathy at the charité) and 1924 (chair for naturopathy at the Friedrich-Schiller University Jena) [31].

A number of naturopathic departments and Kneipp's departments at municipal hospitals were, however, operating further up to 50 s or 60 s when new techniques and new specific medication replaced them.

## 7. Modern Progression of Naturopathy

After the second world war, the naturopathic movement redeveloped in the West and the Kneipp movement with its new concept of five columns was the most successful. In GDR, a part of true naturopathy was integrated into physiotherapy. The Prießnitz hospital, Mahlow, continued its service also.

Presently, there are few establishments in the acute inpatient care sector (Table 1)—in contrast to the outpatient care provided by nonmedical healers and specialized physicians. Complementary and specially naturopathic orientated therapies are practiced within conventional medical hospitals and in rehabilitation hospitals in an increasing scope [32].

Naturopathy is mostly applied by practitioners in the ambulant sector, with about 20.000 physicians specialized in natural healing and about 15.000 nonmedical practitioners in Germany.

In 1968 the “Krankenhaus für Naturheilweisen” at Munich emerged from the former hospital for homoeopathy, which was already founded in 1883, and extended its treatment options to naturopathy [42].

In 1989, Berlin's professorship for true naturopathy was established and located in the Moabit Hospital. Since 1991, the hospital wards are located in the Immanuel hospital at Wannsee [33].

In succession, consistently donation professorships are instituted. There is no government funded professorship yet.

The union of associated hospitals of the “Munich model,” established in 1993, was supported by the Bavarian Ministry of Labour, Family, Social Affairs and Public Health and operated in the first instance until 1999 [43]. The union of hospitals was enlarged by the Dr. Köhler-Parkkliniken, Bad Elster (Saxony) hospital in 1997 (since 2004: German Hospital for Integrative Medicine and Natural Healing). Since 2000, the union of hospitals is named “The Network of Hospitals for Naturally Healing Systems/Complementary Medicine” and is extended by some further hospitals.

In 1995, the Blankenstein hospital in Hattingen applied for the possibility of introducing naturally healing systems in an acute hospital in North Rhine-Westphalia (NRW). With support of health insurance companies the Blankenstein hospital was developed as the first model department. Since 1997, the Blankenstein hospital consists of the Departments of Internal Medicine, Surgery, Anesthesia and Otorhinolaryngology, as well as Model Department of Naturopathy. During 1999–2003, the department was scientifically monitored at the first time [36–38]. In 2005, a further scientific monitoring in association with university hospitals of the Ruhr University of Bochum was implemented to perform an interhospital comparison of naturopathic and conventional inpatient treatment [40]. The running third scientific monitoring concerns the sustainability of the achieved progress of the inpatient stay. The “Ordnungs therapy” is a subject of the evaluation. In an interhospital comparison in the Ruhr area the naturopathic department got better scores for patient satisfaction than the mean of conventional orthopedic departments [39].

In 1999, the second naturopathic model department of the Federal Land NRW was established in the Hospital of Essen, Mitte—the Department “Hospital for True Naturopathy and Integrative Medicine” with 54 beds [41].

Some other naturopathic departments, for example, Hufeland hospital in Bad Ems and Kneipp'sche Kliniken in Bad Wörishofen [44], are developing all over Germany and neighbouring countries, for example, Slotervaart hospital, department of pediatrics, in Amsterdam [45].

## 8. Evaluation of Naturopathy in History

Different ways to describe and evaluate naturopathic treatment in history up to now are delineated in Table 2.

Though naturopathy and conventional medicine were directly compared “side by side” already in the thirties of the last century at Dresden, the published results are academic conversations but no comparable physical or psychological measurements [25, 26]. Comparison of naturopathy and conventional medicine remains difficult. Only one study exists which compares treatment results between naturopathic and conventional orthopaedic, respectively, rheumatologic treatment [46]. Because this study could not randomly assign the patients to the treatment groups, interpretation of the results is difficult. Nevertheless naturopathy seems to be at least as effective as conventional orthopaedic, respectively, rheumatologic treatment. All other modern studies evaluating naturopathic treatment on the whole are one-armed cohort studies without control group.

## 9. Discussion and Conclusions

Naturopathic hospitals have always tried to evaluate their results. Outcome research was a central issue of inpatient naturopathic treatment. Kusche [16] already compared costs of prescribed drugs in 1955, Krauß [27] compared duration of stay and average costs of medication in 1987. The importance of analysing the admitting physicians was already recognized by Kusche [16]. Modern developments of patient-centered health care like “integrierte Versorgung” in Germany emphasize and underline the importance of this type of research. Newer studies use standardized questionnaires to measure the effects of naturopathic inpatient treatment [36–41] allowing for more objective measurement of effects in outcome research. Only one modern study was able to compare naturopathic inpatient treatment with conventional minimal invasive orthopedic, respectively, rheumatologic treatment.

The future development of the inpatient treatment with naturopathy in Germany is uncertain at this time, especially due to discussions on the Law on Modernization of Public Health and decreasing financial resources in public health sector. Also the adopted system of case allowances (DRG-System) complicates the accounting of natural healing treatments in the hospital. The classical naturally healing systems propose ideal prerequisites for prevention and complementary treatment of numerous diseases. Because of the recent political statements considering the development of prevention as one of the central tasks of the future German public health system and implementation of, for example, “the German Forum of Prevention and Facilitation of Public Health” the political intention for maintenance and development of inpatient treatment with naturopathy in Germany may play an important role.

## Figures and Tables

**Table 1:** Overview of the departments providing naturopathic inpatient care today.

Institution	Time period	Treatment approach	Number of beds
Waldhausklinik Deuringen, Stadtbergen	Since 1966	Naturopathy and homoeopathy	40
Hufeland-Klinik, Bad Ems	Since 2000	Naturopathy, homoeopathy, and hyperthermia	40
Klinik für Naturheilverfahren, Akupunktur und Allgemeine Innere Medizin, Krankenhaus St. Josef-Stift, Bremen	Since 2007	Naturopathy, homoeopathy, and traditional Chinese medicine	10
Krankenhaus für Naturheilweisen, München	Since 1883 homoeopathy, since 1966 naturopathy	Naturopathy and homoeopathy	110
Zentrum für Naturheilkunde, Immanuel Krankenhaus, Berlin	Since 1901 in Berlin, Lichtenfelde, since 2001 in Berlin/Wannsee	Naturopathy	40
Klinik für Naturheilkunde u. Integrativer Medizin, Essen	Since 1999	Naturopathy, homoeopathy, and traditional Chinese medicine	63
Abteilung für Naturheilkunde, Klinik Blankenstein, Hattingen	Since 1997	Naturopathy	60

**Table 2:** Evaluation of inpatient naturopathy in history.

Author	Year	Institution analysed	Method
Grote and Brauchle [25, 26]	1935–1938	Rudolf Heß hospital in Dresden	Descriptive dialogues, outlining of methods, and clinical case results

		Naturopathic hospital at Berlin, Lichterfelde (1900)	Historical descriptive, outlining of methods, and analysis of admitting physicians
		Naturopathic hospital at the Charité, Berlin (1920)	
		Naturopathic hospital at Jena university (1924)	
Kusche [16]	1955	Mahlow hospital at Berlin (1927)	
		Rudolf Heß hospital in Dresden (1934)	
		Hospital at Murnau (1932)	
		Department of naturopathy at the Ochsenzoll hospital, Hamburg (1953)	

		Naturopathic hospital at Berlin, Lichterfelde	
Dieckhoefer [31]	1987	Naturopathic hospital at the Charité, Berlin	Historical narrative/descriptive
		Naturopathic hospital at Jena university	

Kühn et al. [33]	1995	Naturopathic department at Moabit hospital/Berlin 1987	Historical description, outlining of therapeutic concepts, and academic teaching

Krauß [27]	1987	Rudolf Heß hospital in Dresden (1934)	Retrospective two-armed cohort study, analysis of duration of stay and average costs of medication

Melchart et al. [34]	1999	Dermatologic Hospital at Höhenkirchen 1994-1995	Prospective, one-armed cohort study, subjective estimation of symptom severity by patients

Melchart and Saller [35]	2002	None	Theoretical concepts

Beer et al. [36–38]	2001-2002	Naturopathic department at Blankenstein hospital, Hattingen	Prospective, one-armed cohort study, standardized questionnairies, statistical evaluation

Beer et al. [39]	2005	Comparison of regional hospitals including Blankenstein hospital, Hattingen, treating orthopedic diseases	Prospective, multicenter, standardised questionnairies and statistical evaluation

		Naturopathic department at Blankenstein hospital, Hattingen	Prospective, multicenter, 3-armed cohort study, standardised questionnairies, statistical evaluation
Wiebelitz et al. [40]	2011	Orthopedic department, St. Josef hospital, university of Bochum	
		Rheumatologic department, St. Elisabeth hospital, university of Bochum	

Lauche et al. [41]	2012	Hospital for True Naturopathy and Integrative Medicine, Essen university	Prospective, one-armed cohort study, standardized questionnairies, statistical evaluation
